# Data Quality and Cost-Effectiveness Analyses of Electronic and Paper-Based Interviewer-Administered Public Health Surveys: Protocol for a Systematic Review

**DOI:** 10.2196/10678

**Published:** 2019-01-30

**Authors:** Atinkut Alamirrew Zeleke, Tolga Naziyok, Fleur Fritz, Rainer Röhrig

**Affiliations:** 1 Division of Medical Informatics Department of Health Services Research Carl von Ossietzky University of Oldenburg Oldenburg Germany; 2 Institute of Medical Informatics University of Münster Münster Germany

**Keywords:** data collection, demographic and health surveys, tablet computers, smartphone, mobile phone, data quality, cost comparison

## Abstract

**Background:**

Population-level survey is an essential standard method used in public health research to quantify sociodemographic events and support public health policy development and intervention designs with evidence. Although all steps in the survey can contribute to the data quality parameters, data collection mechanisms seem the most determinant, as they can avoid mistakes before they happen. The use of electronic devices such as smartphones and tablet computers improve the quality and cost-effectiveness of public health surveys. However, there is lack of systematically analyzed evidence to show the potential impact on data quality and cost reduction of electronic-based data collection tools in interviewer-administered surveys.

**Objective:**

This systematic review aims to evaluate the impact of interviewer-administered electronic device data collection methods concerning data quality and cost reduction in population-level surveys compared with the traditional paper-based methods.

**Methods:**

We will conduct a systematic search on Medical Literature Analysis and Retrieval System Online, PubMed, CINAHL, PsycINFO, Global Health, Trip, ISI Web of Science, and Cochrane Library for studies from 2007 to 2018 to identify relevant studies. The review will include randomized and nonrandomized studies that examine data quality and cost reduction outcomes. Moreover, usability, user experience, and usage parameters from the same study will be summarized. Two independent authors will screen the title and abstract. A third author will mediate in cases of disagreement. If the studies are considered to be combinable with minimal heterogeneity, we will perform a meta-analysis.

**Results:**

The preliminary search in PubMed and Web of Science showed 1491 and 979 resulting hits of articles, respectively. The review protocol is registered in the International Prospective Register of Systematic Reviews (CRD42018092259). We anticipate January 30, 2019, to be the finishing date.

**Conclusions:**

This systematic review will inform policymakers, investors, researchers, and technologists about the impact of an electronic-based data collection system on data quality, work efficiency, and cost reduction.

**Trial Registration:**

PROSPERO CRD42018092259; http://www.crd.york.ac.uk/PROSPERO/display_record.php?ID= CRD42018092259

**International Registered Report Identifier (IRRID):**

PRR1-10.2196/10678

## Introduction

Population-level survey or public health survey is an important method of public health research. It helps monitor sociodemographic events and support policy development and intervention designs with evidence [1,2]. Most developing countries conduct a census or periodic demographic and health surveys to determine national and regional estimates. The population-level epidemiologic indicators help identify the determinants of mortality and morbidity. Although all steps of the data collection and management processes impact data quality, the mechanism of data capture seems to be the main determinant of data quality to avoid mistakes before they happen [1,3,4].

Conducting surveys includes a lot of manual tasks to manage the data collection and reporting processes [5]. Additionally, broader field-based surveys require human and material resources. Inherently, paper data collection processes are labor intensive, time consuming, and susceptible to errors. They incur high printing and running costs and are cumbersome and uncomfortable for the field data collectors [6,7]. The data quality, survey period, and the overall cost of the process can be affected with the above intrinsic nature of paper-based data capturing tools [8,9].

The growth of information and communication technologies such as electronic data collection systems have mitigated some of the challenges encountered in paper-based data collection. Implementation of tablet- or smartphone-based data collection tools is becoming increasingly popular in public health surveys [10,11]. The potential of electronic data collection tools varies according to their intended area of intervention (disease or health care event), country setting, mode of administration (self- or interviewer-administered), and type of research study (clinical trial or survey). A comparison between computer-assisted self-interviews and face-to-face or telephone interviews was conducted in public health studies regarding drug abuse [12] and sexual health and HIV [13-15]. The findings showed that computer-assisted self-interviews were preferable as they resulted in more significant reportings of potentially stigmatized drug, sex, and HIV risks.

Other studies compared paper-based clinical case report forms (CRFs) with electronic CRFs (eCRFs) [16-18]. Electronic data captures (EDCs) were found to be advantageous in broad, low-risk studies and could contribute to improving the data quality and reducing cost. A recent review also showed that the use of EDC in clinical research is cost effective and improves the quality of data [19].

The potential of mobile devices can be seen in demographic health surveys [4,20], general surveys [21], and longitudinal surveys [22]. Studies have proved that electronic data collection tools can improve data quality and work efficiency and reduce overall costs of the survey. However, those studies embedded the impact of the mobile device for data collection in electronic health or mobile health (mHealth) research outcomes. This embedding may compromise the self-standing effect of mobile devices in improving the data collection and management processes of surveys [10,23,24]. Therefore, the impact of electronic data collection tools in surveys needs to be separately analyzed and reported.

A recent review by the Cochrane Collaboration compared the impact of apps and alternative instruments such as paper, laptop computers, and tablet computers for self-administered health surveys [11]. Data collection process involves an interaction between the questionnaire, the respondent, and, in the case of interviewer-administered surveys, the interviewer. In contrast with self-administered surveys, during the interviewer-administered survey, the interviewer or data collector is an additional mediating factor in the interactions of interview tools and the respondents. The difference in the mode of questionnaire administration can have serious effects on data quality [9]. A systematic review considering interviewer-administered data collection may complement that evidence. As to the knowledge of the investigators, there is no systematic review that has analyzed data quality and cost-effectiveness of electronic and paper-based interviewer-administered public health surveys. This systematic review will fill this gap by answering the following question for interviewer-administered public health surveys: What evidence is available for the differences in data quality and cost-effectiveness between electronic and paper-based capture of data?

## Methods

### Study Registration

The protocol is registered in the International Prospective Register of Systematic Reviews (CRD42018092259). This protocol follows the Preferred Reporting Items for Systematic Review and Meta-Analysis Protocols 2015 guideline [25].

### Eligibility Criteria

We have categorized the inclusion criteria for this systematic review according to study design, study participants, types of intervention, types of technology, and study setting.

### Study Design

We will include parallel randomized controlled trials (RCTs), quasi-RCTs, controlled clinical trials, crossover RCTs, paired repeated measures, cohort and case-control studies, and comparative cross-sectional studies that compare the electronic interviewer-administered survey with paper-based methods.

### Study Participants

This review focuses on data quality outcomes from the data collected by data collectors who used paper or electronic data collection modalities during public health surveys. We will also include data collectors, supervisors, or data managers for opinion-, preference-, and usability-related analysis.

### Types of Intervention

Any mobile device data collection tool that was designed to support interviewer-administered data collection processes in public health surveys will be included.

### Types of Technology

Electronic data collection in our review refers to portable, wireless digital devices usually supported by mobile network or satellite communication infrastructures, such as cell phones, smartphones, personal digital assistants, and tablet computers. The given support includes data capture and instant, stored, and forward transfers to the research center. We will include all apps with technologies that directly support the data collection process by enabling data collectors or interviewers to collect and send data as well as enabling supervisors or data managers to monitor the data collection process.

### Study Setting

Our review will include national demographic surveys, demographic and health surveillance systems, and household surveys. We will include all countries and research facilities regardless of the socioeconomic status of the country.

### Exclusion Criteria

We will exclude the following study types from the review: all studies that compare electronic and paper-based tools in self-administered surveys; studies that are performed in settings other than a house-to-house field survey (eg, electronic medical records and eCRFs); studies not performed on human subjects; studies reported before January 1, 2007; studies that are experience reports, letters, reviews, commentaries, and editorials; and non-English language publications.

### Outcomes

The primary outcomes in this review will be data quality indicators and cost-effectiveness evidence. According to Bowling [9], data quality is a vague concept, and it is hard to find any gold standard or framework. Data quality could be defined in terms of survey response rates, questionnaire item response rates, the accuracy of responses, the absence of bias, and completeness of the information obtained from respondents [9]. For this review, we will focus on two data quality indicators: data completeness and data accuracy. Accuracy is hereby defined as the absence of typographical errors, decimal point faults, and illogical values, whereas the completeness of items is inversely proportional to the number of missing responses in the questionnaire. In general, we will compare the proportion of errors or missing items between electronic and paper-based data collection methods [9,11,26,27]. Cost-effectiveness outcomes will be measured using resource costing methods, which include provider perspective direct cost comparing the cost of conducting a survey using electronic and paper-based data collection methods. The secondary outcomes include work efficiency, usability, user experience, and acceptability.

### Information Source

We will conduct a systematic keyword search on electronic databases such as Medical Literature Analysis and Retrieval System Online, PubMed, CINAHL, PsycINFO, Global Health, Trip, ISI Web of Science, and Cochrane Library. In addition, we will screen the reference list citations of included articles. Unpublished and in-progress studies will be identified from the following trial registries: ClinicalTrials.gov, ISRCTN registry, Australian New Zealand Clinical trial Registry, International Clinical Trials Registry Platform.

We will restrict our search to articles published in English from 2007 to mid-2018 (as mobile devices that became available during this time are compatible with the mobile operating system framework that focuses on apps, and most of the EDC apps were tested during this period [11]).

### Search Strategy

The search strategy will consider 3 categories: the technology or intervention used (eg, mobile device, mobile phone, mHealth, or EDC), area of application (eg, data collection, demographic and health surveys, or large-scale surveys), and the outcome of interest (eg, data quality, missing data, and cost-effectiveness). We will connect all the similar terms in the same group with the Boolean operators “OR” and “AND” (Table 1).

### Data Management

Endnote software (Clarivate Analytics) will be used to import the retrieved literature from all databases to manage duplication and further screening. The Covidence (Veritas Health Innovation) Web-based screening tool will be used to import the set of deduplicated citations and to manage the title and abstract screening process. We will screen the titles and abstracts for the inclusion criteria. Potentially relevant full-text papers will be reviewed, including reference lists of these papers.

### Selection Process

Two authors (AAZ and TN) will screen titles and abstracts independently and identify potentially eligible studies based on the eligibility criteria. Review author pairs will then screen the full-text reports and decide whether these meet the inclusion criteria. Any disagreements in each phase will be resolved first by discussion among the review authors using the prespecified inclusion criteria. If the disagreement or uncertainty continues, a consulting third author (FF) will mediate the final decision. We will note the reasons for inclusion and exclusion using a flowchart diagram.

### Data Extraction

An excel sheet for data extraction will be used based on the inclusion criteria and the objectives of the review. To ensure uniformity across reviewers, we will conduct a pretest standardization exercise before starting the data extraction process. Textbox 1 presents the data items that we will extract.

**Table 1:** Search terms and preliminary search results from PubMed and Web of Science searched in March 2018.

#	Category	Terms	PubMed	Web of Science
1	Technology or intervention	(mobile phone OR cellular phone OR Cell Phone OR cellphone OR smart phone OR smartphone OR tablet device OR Tablet Computers OR Computers, Handheld OR Computer, PDA OR personal digital assistant OR electronics data capture OR EDC OR electronic survey OR eCRF OR electronic forms OR eHealth^a^ OR mHealth^b^ OR Mobile Technology OR Mobile Application OR Mobile Apps OR App, Mobile OR Apps)	111,669	125,598
2	Area of application	(Data collection OR electronic data collection OR electronic data capture OR Paper-Based data collection OR data entry OR data capture OR data gathering OR questionnaires OR survey OR health survey OR interview OR demographic OR household survey OR large-scale surveys OR population surveillance OR Demographic Health Survey OR DHS^c^)	3,248,226	2,601,782
3	Outcome of interest	((Cost Analysis OR Cost Comparison OR cost saving OR Costs OR Cost Measures OR Cost-Benefit Analyses OR Cost Effectiveness OR Cost-Utility Analysis OR Economic Evaluation) AND (Data quality OR Data accuracy* OR error OR error rate OR missing OR incompleteness OR inaccuracy))	24,247	67,272
Combined		1 AND 2 AND 3	1491	979

^a^eHealth: electronic health.

^b^mHealth: mobile health.

^c^DHS: demographic health survey.

**List of the data items that will be extracted based on the inclusion criteria and the objectives of the review.**

- Author and year
- National affiliation of the author
- Country in which the study was conducted
- Study design
- Health care or research site setting
- Target users
- Size of enumerated population dataset or data elements
- Type of mobile device, delivery mode, app type
- Stated purpose of intervention
- Range of data quality outcome measures described based on our operational definition for data quality parameters
- Range of economic evaluation outcomes used to evaluate the cost-effectiveness
- Types of economic evaluation models or outcomes assessed
- Usability, user experience, and work efficiency outcomes or descriptions
- Key findings from each included study will be summarized and tabulated

### Outcomes and Prioritization

The primary outcomes of this review are the following data quality indicator parameters: error rates quantified from missing items (number of incomplete records per interview questionnaire) and inaccuracy (mean number of problematic records per interview questionnaire). The secondary outcomes are cost-effectiveness parameters and cost-related outcomes. Moreover, we will consider usability scale and qualitative user satisfaction indices for the secondary outcomes analysis. Multiple definitions of usability, acceptability, user satisfaction, and related terminologies exist [11]. We will extract, summarize, and categorize the definitions in the final filtered full-text papers to include efficiency, acceptability, usability, and user experience outcomes.

### Risk of Bias in Individual Studies

The quality of the included studies will be assessed using parameters such as random sequence generation, allocation concealment, blinding of participants and personnel, blinding of outcome assessment, incomplete outcome data, selective reporting, and other biases. We will grade each parameter as a low, high, or unclear risk of bias [28]. All assessments of study quality will be performed by at least two reviewers (AAZ and TN), with any disagreement resolved by consensus or mediation with a third reviewer (FF or RR) where necessary.

### Data Synthesis

We will present the analyzed data in a tabular and narrative form. Where possible, meta-analyses will be performed on methodologically comparable studies (comparable particularly with regards to the study design and endpoint measures in the outcomes) reporting primary and secondary outcomes. The choice of statistical tests will depend on the nature of the outcome variable. Where relevant data are missing, we will contact the authors. If we cannot obtain missing data by contacting the authors, we will use an imputation method. If the number of included studies per outcome is sufficient, publication bias will be assessed visually through funnel plots and tested by Egger’s regression test. The Mantel-Haenszel method will be used for the fixed effect model if tests of heterogeneity are not significant. If our data displays statistical heterogeneity, the random effects model will be selected. In cases of significant heterogeneity, we will perform a qualitative narrative summary instead of a meta-analysis.

### Ethics and Dissemination

As only previously published studies are included and reported in the review, no additional formal ethical assessment and no informed consent is required. The findings will be disseminated through publication of a single manuscript in a peer-reviewed journal.

## Results

We anticipate January 30, 2019, to be the finishing date.

## Discussion

This systematic review will identify and synthesize the available evidence on data quality and cost-effectiveness outcomes of electronic data collection tools for interviewer-administered surveys. The evidence from the systematic review is supposed to complement the available evidence on the impact of mHealth on demographic and health care data collections [29].

## Abbreviations

CRF: case report form
eCRF: electronic case report form
eHealth: electronic health
EDC: electronic data capture
mHealth: mobile health
